# Influence of Age and Neurotoxic HAART Use on Frequency of HIV Sensory Neuropathy

**DOI:** 10.1155/2012/961510

**Published:** 2012-04-17

**Authors:** Olajumoke Oshinaike, Akinsegun Akinbami, Oluwadamilola Ojo, Anthonia Ogbera, Njideka Okubadejo, Frank Ojini, Mustapha Danesi

**Affiliations:** ^1^Department of Medicine, Lagos State University College of Medicine, Lagos State, Nigeria; ^2^Department of Hematology and Blood Transfusion, Lagos State University College of Medicine, Lagos State, Nigeria; ^3^College of Medicine, University of Lagos, Idi-araba, Lagos State, Nigeria

## Abstract

*Background*. Sensory neuropathy (SN) is one of the most common AIDS-associated neurologic disorders especially in the era of highly active antiretroviral therapy (HAART). The aim of this study was to determine the prevalence of SN among highly-active-antiretroviral-therapy- (HAART-) experienced and HAART-naïve HIV-positive individuals and to investigate the relationship to demographic, clinical, and laboratory factors. *Methods*. 323 patients with HIV infection (142 on HAART and 181 HAART naïve) were enrolled in a cross-sectional neuropathy screening program. Data was collected using structured questionnaires which contained the brief peripheral neuropathy screening tool of AIDS Clinical Trial Group protocol. Neuropathy was defined by the presence of at least 1 clinical sign in a distal, symmetrical pattern. Patients were classified as symptomatic if they described aching, stabbing, or burning pain, paresthesia, or numbness in a similar distribution. Demographic, clinical, and laboratory details were documented as risk factors. *Result*. The prevalence of sensory neuropathy was 39.0% (126/323), (of which 29/126 (23%)) were symptomatic. Amongst those on HAART, 60/142 (42.3%) had SN compared to 66/181 (36.5%) HAART-naïve individuals (*P* = 0.29). On multivariate analyses, the independent associations with SN were increasing age (*P* = 0.03) and current exposure to stavudine (*P* = 0.00). Gender (*P* = 0.99) height (*P* = 0.07) use of HAART (*P* = 0.50), duration of HAART treatment (*P* = 0.10), and lower CD4 count (*P* = 0.12) were not associated with an increased SN risk. *Conclusion*. HIV SN remains common despite improved immunologic function associated with HAART and decreased neurotoxic HAART use. In this cross-sectional analysis, age and stavudine-based therapies were the independent risk factors.

## 1. Introduction

Peripheral neuropathy is common at all stages of human immunodeficiency virus (HIV) infection and causes considerable morbidity. The manifestations include focal mononeuropathies (particularly cranial neuropathies) polyneuropathies such as the inflammatory demyelinating polyneuropathies (acute, subacute, and chronic subtypes), and sensory neuropathy, which is the most common. In the pre-HAART era, HIV sensory neuropathy (HIV-SN) was documented in as high as 35% of patients with AIDS [1]. With the advent of HAART, the prevalence has increased, a situation partially attributable to the emergence of antiretroviral-induced toxic neuropathy, particularly with the nucleoside analogue reverse transcriptase inhibitors (NRTIs) [2, 3].

Toxic or drug-induced sensory neuropathy is clinically indistinguishable from sensory neuropathy of other aetiologies encountered in the setting of HIV. However, aetiological clues include a recent history of drug implementation, improvement after drug withdrawal, and elevated serum lactate concentration [4]. NRTIs used in antiretroviral therapy may cause mitochondrial toxicity and dysfunction which leads to the disturbance of the glucose metabolism, resulting in an accumulation of lactate. The pathogenesis of sensory neuropathy in HIV remains debatable, although the viral neurotoxicity hypothesis is favored. Indirect neuronal damage is thought to occur as a consequence of immune activation that leads to the release of cytokines, free radicals and lipid membrane derivatives which are potentially neurotoxic. In ARV-associated sensory neuropathy, mitochondrial damage is implicated, with dysfunction attributed to the inhibition of DNA polymerase gamma, the enzyme responsible for mitochondrial DNA replication. Chemotherapeutic agents used to treat AIDS-related conditions such as vincristine, isoniazid, and hydroxyurea can also cause HIV-SN [5]. The clinical presentation of HIVSN is marked by symmetrical often burning painful paraesthesias in the feet, which may be so severe as to cause contact allodynia and difficulty with mobility. Involvement of the upper extremities and distal motor weakness may occur later in the course. Diagnostic modalities include the clinical examination and electrodiagnostic evidence of predominantly axonal neuropathy [6]. Sural nerve biopsy shows degeneration of myelinated and unmyelinated axons with necrotizing vasculitis [7]. Skin biopsies with intraepidermal nerve fibre density determination can identify patients with sensory neuropathy and can be used as an outcome measure. Treatment is largely unrewarding, and partial symptomatic relief is obtained from pain-modifying agents such as nonsteroidal anti-inflammatory drugs, antidepressants, anticonvulsants, or, in severe cases, narcotic analgesics. ARV therapy may improve sensory function in less advanced disease [8, 9].

This study was designed to estimate the burden of sensory neuropathy in HIV/AIDS (acquired immunodeficiency syndrome) patients and exploring the disease-related and treatment-related determinants of its occurrence.

## 2. Methods

### 2.1. Study Population

This is a cross-sectional study conducted at the HIV clinic of the Lagos State University Teaching Hospital over a one-year period from January to December 2010. The study enrolled patients attending the HIV clinic, who were at least 18 years and satisfied the World Health Organization (WHO) clinical case definition for HIV/AIDS. Eligibility criteria included the ability to undergo a clinical interview consisting of a standardized neurological evaluation for symptoms and signs of HIV-SN. Individuals were excluded if they were on antituberculous medication, had diabetes mellitus, chronic kidney disease, or active opportunistic infections or did not give consent. The hospital ethics committee approved this research, and each participant gave informed consent.

### 2.2. Methods

The demographic characteristics and information regarding HAART use were obtained for all participants. This was followed by the administration of the AIDS clinical trial group (ACTG) Peripheral Neuropathy Screening Tool. The instrument has been used widely in clinical trials and encompasses both symptoms and clinical signs suggestive of neuropathy. It has been shown in a large cohort of individuals with HIV infection to detect accurately those who have the greatest degree of abnormality on formal sensory threshold testing. HIV-SN was diagnosed in the presence of at least 1 sign bilaterally (in consonance with the CHARTER study, to enable higher sensitivity for detection of SN) [10]. Patients were classified as symptomatic if they described aching, stabbing, or burning pain, paraesthesia, or numbness in a similar distribution. The presence of potential clinical correlates and risk factors for HIV-SN such as patients height, HAART use, including current exposure to specific HIV-SN-associated drugs (stavudine-based HAART), alcohol abuse/dependence, and HIV disease marker (CD4 cell count) were also evaluated. Prior exposure to stavudine-based therapies was not ascertained.

### 2.3. Data Analysis

Data were analysed using SPSS version 17.0 statistical package. Continuous variables such as participant's age, height, and CD4 cell count are presented as mean ± standard deviation (SD), and comparison between groups was made using analysis of variance. Categorical variables (e.g., gender, CD4 cell count category below or above 200 cells/mm^3^, HAART group) are presented as frequencies (%) and group differences compared using nonparametric Yates' corrected *X* ^2^ test. Multivariate analysis was applied to determine the independent predictors of occurrence of HIV-SN.

## 3. Results

### 3.1. Demographic Characteristics

323 participants comprising 218 (67.5%) females and 105 (32.5%) males were studied. 97 (68.3%) females and 45 (31.7%) males were on HAART (total 142) whilst 121 (66.9%) females and 60 (33.1%) males were HAART naïve (total 181). There was no significant gender difference between the groups (*X* ^2^ = 0.77; *P* = 0.81). The mean age of the HAART-experienced group was 38.3 ± 10.8 (median 35.5), while that of the HAART-naïve controls was 35.5 ± 8.7 (*P* = 0.009). Mean CD4 cell count (cells/mm^3^) was 246.1 ± 152.2 in the HAART group and 189.7 ± 150.1 in the HAART-naïve group (*P* = 0.001) Table 1. The HAART group was characterized by specific drug combination into two groups: stavudine based (12/142) and non-stavudine based (130/142). The duration of exposure to HAART was 1–48 months, with a mean of 16.4 ± 10.7 months (median 17.0 months).

### 3.2. Prevalence and Risk Factors of HIV-Associated Sensory Neuropathy (HIV-SN)

HIV-associated sensory neuropathy, defined as the presence of at least 1 abnormal neuropathy sign, was present in 126 (39.0%) of the entire cohort (*N* = 323). Of the patients with HIV-SN, 29 (23.0%) were symptomatic, while 97 (77.0%) were asymptomatic. The frequency of HIV-SN in the HAART-experienced group was 42.3% (60/142), whilst it was 36.5% (66/181) in the HAART-naïve group (*P* = 0.29). The clinical characteristics of the HIV/AIDS patients with and without sensory neuropathy are shown in Table 2.

The determinants of occurrence of HIV-SN were explored using variables obtained in this study. Specifically, the effect of age group (above 40 years), gender, height (above 1.7 m), HAART use, duration of HAART use (above 12 months), and ARV category (stavudine or non-stavudine-based therapy) was evaluated in multivariate logistic regression analysis. Age above 40 years (*P* = 0.03) and stavudine-based ARV use (*P* = 0.00) were significantly independently associated with the occurrence of sensory neuropathy, the strongest determinant being the latter (Table 3).

## 4. Discussion

This cross-sectional study on the burden of HIV-sensory neuropathy has added to existing body of evidence that HIV-SN occurs frequently in people with HIV/AIDS, with a frequency of 39% overall and 36.5% on cases on HAART. Recent estimates of HIV-SN prevalence among cohorts with access to HAART ranges from 20 to 50% irrespective of whether there is additional risk such is conferred by the use of established neurotoxic antiretroviral drugs [11, 12]. Also, available evidence suggests that HIV-SN prevalence remains high among HAART-treated patients, even in regions where known neurotoxic antiretroviral drugs such as stavudine are no longer commonly used [12]. In this study, only 12/323 cases were on stavudine-based HAART suggesting that other risk factors should be considered and the aetiology of HIV-SN is clearly beyond that attributable to use of neurotoxic HAART. Host genotype has been associated with HIV-SN risk among neurotoxic HAART-exposed patients in several groups. Consistent with pathogenic mechanisms of HIV-SN, the associations include mitochondrial haplogroups and genes associated with inflammation. Associations with hemochromatosis gene polymorphisms have also been reported [13, 14].

Considering the persisting risk of HIV-SN despite optimizing therapy for HIV, we explored the determinants of occurrence of HIV-SN and found one nonmodifiable independent predictor, that is, advancing age above 40 years, and most importantly, a modifiable risk factor, that is, the use of stavudine-based therapy. Gender, height, lower CD4 count, use of HAART, and duration on HAART were not associated with increased risk. Ellis in the Central Nervous System HIV Antiretroviral Therapy Effects Research (CHARTER) study [10] had documented lower nadir CD4 count and the use of HAART, in addition to advancing age, as risk factors. He speculated that HAART is neurotoxic as it was associated with higher SN prevalence. Maritz and colleagues also documented use of HAART in addition to age as risk factors of SN.

Age has remained a consistent independent risk factor in almost every published cohort, both before and after the introduction of HAART [15–17]. This is consistent with the known vulnerability of the aging peripheral nervous system to most types of polyneuropathies, HIV-SN inclusive. Height has also been identified as a risk factor for sensory neuropathy in HIV infection on HAART. The pathology involves a length-dependent degeneration of both small and large peripheral nerve fibers making taller individuals more prone to the development of SN. Cherry et al. [18] had documented that the risk of HIV-SN was 65% higher among the older (>40 years) and taller (>170 cm) HIV patients exposed to stavudine. They proposed that age and height measurements represent an effective way of identifying individuals at higher risk for neuropathy and prioritizing them to alternative HAART therapy rather than neurotoxic HAART. Height was not found to be an independent risk factor in this study probably due to the small number of patients that were above 1.7 m.

Previously reported literature assessing immune status and HIV-SN is mixed, with some reports linking neuropathy to increased viral load levels, while others have reported no relationship. Several studies from the highly active antiretroviral therapy (HAART) era show a lack of association between distal painful sensorimotor polyneuropathy and the degree of immunosuppression, including low CD4 counts and high HIV viral load [19, 20]. Morgello and colleagues speculate that this difference in findings may relate to a difference in patient populations. Other reasons may include residual axonal injury even with restoration of immune function, immune reconstitution disorders or even the presence of other confounding neurotoxins such as nutritional or vitamin deficiencies. Also the association between neuropathic pain and a higher CD4 nadir may suggest that a functional immune system may contribute to the stimulation of pain.

Limitations of our study include a cross-sectional design as a longitudinal follow-up would provide further insight into the incidence of HIV-SN in Nigerians. Furthermore, the nonavailability of electrophysiological studies limited diagnostic accuracy.

## 5. Conclusion

This study has confirmed the high prevalence of SN in HIV/AIDS patients on HAART. SN persists despite improved immunologic function associated with HAART and decreased neurotoxic HAART use. With these high prevalent rates and majority experiencing debilitating neuropathic pain, HIV-SN represents a huge and potentially worsening source of world morbidity. There is need to develop more effective preventative strategies and treatments to control the symptoms associated with existing cases of HIV-SN.

## Figures and Tables

**Table 1:** Demographic and clinical characteristics of 323 participants in the HIV-SN study.

Variables	All *N* = 323	HAART treated *N* = 142	HAART naïve *N* = 181	*P* value
Male	105 (32.5%)	45 (31.7%)	60 (33.1%)	0.81
Female	218 (67.5%)	97 (68.3%)	121 (66.9%)	
Mean age ± SD (years)	36.7 ± 9.8	38.3 ± 10.8	35.5 ± 8.7	0.009
Age category >40 years	9 (30.0%)	51 (52.6%)	48 (49.5%)	0.09
Mean height ± SD, metres	1.61 ± 0.08	1.59 ± 0.07	1.62 ± 0.09	0.000
Height category >1.7 metres	44 (13.6%)	4 (2.8%)	40 (22.1%)	0.000
Mean CD4 count ± SD, cells/mm^3^	214.5 ± 153.4	246.1 ± 152.2	189.7 ± 150.1	0.001
CD4 count >200, cells/mm^3^	147 (45.5%)	81 (57.0%)	66 (36.5%)	0.000
CD4 count ≤200, cells/mm^3^	176 (54.5%)	61 (43.0%)	115 (63.5%)	

**Table 2:** Relationship between demographic and clinical variables and the occurrence of HIV-sensory neuropathy.

Variables	SN present *N* = 126	SN absent *N* = 197	*P* value
Mean age ± SD, years	38.2 ± 10.6	35.8 ± 9.1	0.03
Male (*N* = 105)	48 (45.7%)	57 (54.3%)	0.09
Female (*N* = 218)	78 (35.8%)	140 (64.2%)	
Mean height ± SD, metres	1.62 ± 0.08	1.60 ± 0.08	0.15
HAART treated (*N* = 142)	60 (42.3%)	82 (57.7%)	0.29
HAART naïve (*N* = 181)	66 (36.5%)	115 (63.5%)	
Mean duration of HAART ± SD, months*	15.9 ± 9.4	16.9 ± 11.6	0.59
Stavudine-based HAART (*N* = 12)	10 (83.3%)	2 (16.7%)	0.003**
Non-stavudine-based HAART (*N* = 130)	50 (38.5%)	80 (61.5%)	
Mean CD4 count ± SD, cells/mm^3^	180.9 ± 138.2	236.0 ± 159.0	0.002
Median CD4 count ± SD, cells/mm^3^	165.5	185.0	
CD4 count ≤ 200, cells/mm^3^ (*N* = 176)	74 (42.0%)	102 (58.0%)	0.22
CD4 count >200, cells/mm^3^ (*N* = 147)	52 (35.4%)	95 (64.6%)	

*Duration of therapy for the 142 participants on HAART therapy. **Fisher's exact *P* value.

**Table 3:** Determinants of occurrence of HIV-sensory neuropathy using multivariate regression analysis.

Variable	*B*	Standard error	*P* value
Age (< or ≥40 yrs)	−.566	.271	0.03*
Gender	−.004	.293	0.99
Height (< or ≥1.7 m)	.627	.353	0.07
CD4 category (≤ or >200)	.384	.246	0.12
HAART use	.257	.389	0.50
HAART type, (stavudine/Non-stavudine-based)	−2.525	.833	0.00*
Duration of HAART use, (< or >12 months)	.666	.409	0.10

*Significantly associated with the presence of HIV-SN.
